# Multi-Target Tracking of Human Spermatozoa in Phase-Contrast Microscopy Image Sequences using a Hybrid Dynamic Bayesian Network

**DOI:** 10.1038/s41598-018-23435-x

**Published:** 2018-03-22

**Authors:** Abdollah Arasteh, Bijan Vosoughi Vahdat, Reza Salman Yazdi

**Affiliations:** 1 Department of Electrical Engineering, Sharif University of Technology, Tehran, Iran; 2 Department of Andrology, Reproductive Biomedicine Research Center, Royan Institute for Reproductive Biomedicine, ACECR, Tehran, Iran

## Abstract

Male infertility is mostly related to semen and spermatozoa, and any diagnosis or treatment requires the investigation of the motility patterns of spermatozoa. The movements of spermatozoa are fast and involve collision and occlusion with each other. In order to extract the motility patterns of spermatozoa, multi-target tracking (MTT) of spermatozoa is necessary. One of the most important steps of MTT is data association, in which the newly arrived observations are used to update the previous tracks. Dynamic Bayesian network (DBN) is a powerful tool for modeling and solving various types of problems such as tracking and classification. There can also be a hybrid-DBN (HDBN), in which both continuous and discrete nodes are present. HDBN has a suitable structure for modeling problems that have both discrete and continuous parameters like MTT. In this research, the data association for MTT of human spermatozoa has been studied. The proposed algorithm was tested over hundreds of manually extracted spermatozoa tracks and evaluated using several standard measures. The superior results of the proposed algorithm in comparison to the other well-known algorithms, show that it could be considered as an improved alternative to traditional computer assisted sperm analysis (CASA) algorithms.

## Introduction

Statistics show that infertility is a problem for many couples. In every four couples, on average, one couple is affected by infertility in developing countries^1,2^. In the majority of cases, the infertility of men has a relationship with spermatozoa and semen, and can be measured by semen and spermatozoa analysis for more advanced diagnosis and treatments^3–6^. Nowadays, many of these analyzes are performed using computer-based systems called computer assisted sperm analysis (CASA). The CASA is a device that consists of software and hardware parts which monitor and measure many kinematic parameters of spermatozoa such as speed, average path, curvature of the path, total movement, etc. All the aforementioned parameters are extracted with the aid of a post process on spermatozoa tracks. The accuracy of the measured parameters is directly affected by the accuracy of each spermatozoon track extraction. Hence, the main problem here involves multiple-target tracking (MTT) to extract the tracks of the spermatozoa. Most of the current CASA algorithms are based on simple methods that were first developed in the past decades and may fail in complex situations like high density samples^7^. There are many MTT algorithms developed and applied to solve many problems such as human tracking^8^, visual object tracking^9^, stem cell tracking^10^, spermatozoa tracking^11^, etc., but spermatozoa tracking is a special problem that should be solved in its appropriate way. Fast nonlinear movements, high density of occlusions, and brightness changes in the image sequences are some of the circumstances that exist in the MTT of the spermatozoa.

There are many studies focusing on the estimation of spermatozoa movement parameters from a few decades ago^12–14^. Some studies focus on single cell tracking^14–16^ and the others concentrate on multiple cell tracking^11,17,18^. It is obvious that tracking multiple cells at once is harder than tracking just a single cell. Thus, the MTT approach is much more useful because extracting many population properties, such as average speed, requires the tracking of many cells at once, and then, a computation of the mean speed, and its reporting for the physician’s diagnosis. In Sørensen *et al*. paper^11^, the core part of the algorithm is the Particle Filter combined with Kalman Filter. The segmentation algorithm is a scale-space blob detector and the final detection rate is not reported. There are no well-known metrics such as F1 measure reported and the reported results are marked “approximately” without any more details on the precision of that approximation in Table 1, and there is no comparison with the other methods. Finally, the studied number of video sequences and tracks are very few (3 video sequences and “approximately” 97 sperm tracks) in comparison to the current study (36 video sequences and 1659 sperm tracks).

**Table 1:** Recorded dataset properties.

Dataset property	Value
Number of image sequences	36
Minimum spermatozoa cell count	4
Maximum spermatozoa cell count	96
Average cell count (rounded)	46
Total number of spermatozoa tracks	1659

MTT of spermatozoa is performed in CASA systems and many parameters are extracted from detected tracks, e.g., curvilinear velocity, straight line velocity, and mean angular displacement, etc.^19^. Spermatozoa motions can be categorized in three motility classes according to the World Health Organization (WHO); these are: progressively moving, non-progressively moving, and immotile^19^. The population of each class in the final reports of a CASA system is very important for later diagnosis and treatments; thus, the important point in the overall process is to accurately track the spermatozoa in image sequences.

MTT has numerous applications and many algorithms have been developed for performing this task^10,15,20–22^. Developing a solution for MTT depends on the details of the problem that have to be solved^23^, but in the most general case, a varying number of targets move on a background, and there are observations which give a noisy data from the targets positions^24^. The noisy observation of the target position means that the detection probability does not equal to one, and that there is always an error in detecting targets. There is also another kind of error in the observations: detecting non-targets as real targets, which are called False Alarms or Clutters^24^.

MTT has to accomplish three main tasks; these are: (1) observation (2) data association, and (3) state estimation. The most important step in MTT is the second step, i.e., the data association^24,25^. The current study did not focus on the first step. The third step is also straightforward after performing the second step and can be performed based on the chosen dynamics and observation models^26^. The main focus of the current study is the second step.

Data association refers assigning the next step’s observations to the current step’s tracks; thus, every observation in the next frame will be assigned to at most one track in the current step and no more than one track will be allowed to be assigned to every observation in the current step. The final output of the data association is the set of all the observations labeled as separate tracks and clutters.

If the targets do not have a distinguishable feature (as in the spermatozoa-tracking) like color, size, shape, etc., the tracking task will be the hardest step as between the current frame and the next, there will be *O*(*n*!) possible data associations (permutations), in which *n* is the number of detections in the current frame. If the sampling frequency of a video sequence is enough, then the number of detections will be close together in consequent frames. It means that if we have *n*etections in frame *t* and *m* detections in frame *t* + 1, then *m* would be in the order of *n* (not necessarily equal to *n*, so the time complexity would be *O*(*n*!). Initially, the data association is an NP-Hard task^27^. Many developed algorithms try to solve the problem faster by making an extra hypothesis, or removing some low-probable hypothesis or gating^21^; some algorithms choose other heuristic approaches to overcome this problem^20^.

One well-known algorithm for solving this data association is the multiple hypothesis tracker (MHT), which is essentially a maximum a posteriori probability (MAP) estimator^23^. In this algorithm, certain hypotheses are formed in each step and as new observations arrive, new hypotheses are formed based on the previous hypotheses, and the output is a hypothesis with the maximum a posteriori probability. However, the computational complexity of MHT algorithm is high^23^ because of the exponential growth of the number of hypotheses as the algorithm progresses in time, but several heuristic methods have been developed for dealing with this problem such as gating^20^ or k-best hypotheses^21^, yet the result is a suboptimal solution.

Another method that has been applied for solving a variety of MTT problems is the joint probabilistic data association filter (JPDAF) which is the generalization of probabilistic data association (PDA). This method approximates each target state as an independent random variable with a Gaussian PDF^28^. This algorithm assumes that the number of targets is fixed and cannot start a new track or end a track in a specific step of tracking^29^. JPDAF is a suboptimal solution for the MTT problem because it approximates the conditional PDF of the target’s state at every stage^28^.

Many other algorithms have been developed like the Nearest Neighbor Filter (NNF)^30^, which is a heuristic greedy method and assigns new observations to the closest predicted position of previously detected tracks, or the Markov Chain Monte Carlo methods^26^, which have their own disadvantages such as a high rejection rate^31^, or other sampling based methods like Gibbs sampling or Particle Filtering, which have been developed for general purpose tracking.

Bayesian Networks (BN)^32^ utilize a graphical structure for the representation of direct dependencies between variables. Dynamic Bayesian Networks (DBN)^33^ are like BNs, but the parameter of time is also involved in them so it can model the dynamics of the systems. DBNs support the modeling of discrete systems in a convenient and compact way. DBNs also support models that include both discrete and continuous variables called hybrid models^34^. Hidden Markov Models (HMM) and Kalman filters are well-known special cases of DBNs^35^.

DBNs are powerful tools for modeling and solving many types of problems such as vehicle classification^36^, tracking hand for hand gesture recognition^37^, human body model, and tracking based on a figure and articulated model^38^. There are other applications of DBNs in modeling dynamic systems, especially in object tracking^39,40^. The most important problem of DBNs is to make an inference based on some evidence, which, initially, needs exponential time in the number of nodes to be computed^41^.

The quantitative relationship between one node in the DBN model and its parents consists of conditional probability distribution (CPD), which defines a conditional distribution for a node based on its parents’ configurations^34^. CPDs are often defined as a table in fully discrete DBN (DBN that all of its nodes are discrete). There can also be hybrid-DBN (HDBN), in which both continuous and discrete nodes are present, e.g., a continuous Gaussian node *X* with discrete parent *U* can be represented as a conditional Gaussian^34^. If a continuous node has continuous parents, the linear Gaussian model would be formed; on the other hand, if a continuous node has both discrete and continuous parents, a model which is called Conditional Linear Gaussian (CLG) would be the dependency model^34^.

The final case is a discrete child with continuous parents. Softmax density^42^ is a suitable model for this case. Softmax CPD^43^ defines the *R*egions by a set of *R* linear functions over continuous variables. Choosing an arbitrarily large *R* for each problem is the key to the power of generalized Softmax CPD, which have been used in this study for building a suitable HDBN model to solve the MTT problem, exploiting the manually extracted dataset (ground-truth) of recorded image sequences.

The main contribution of the current study is in the usage of the manually extracted dataset under an adapted formulation of Softmax CPD in a novel HDBN structure that solves the data association problem, and automatically starts and ends a varying number of tracks. The proposed structure yields better results in comparison to the other well-known methods. Achieving better results compared to the other well-known methods is the other contribution; for reaching those results, however, two important contributions in developing the algorithm have been made. The first contribution involves the utilization of graphical models and HDBN for solving the data association; for this, a new approach was developed for adapting the Softmax CPD to the data association problem in an appropriately designed HDBN. Secondly, gating was used to reduce the hypotheses space by removing hypotheses with low probabilities for making the inference feasible in the designed HDBN network. With this approach, the computational complexity of the algorithm is a function of the size of the reference manually extracted dataset and the gated hypotheses set. It is also well worth mentioning that the dataset of this study is quite large and has 36 image sequences and a variety of cell counts, ranging from 4 to 96, while many other methods use achieved good results in less than 10 cells in reported results^6^ or used very few sample sequences (just two sequences in^7^). The dataset of the current study consists of 1,659 cell tracks.

## Methods

### Data Acquisition

**Figure 1:**
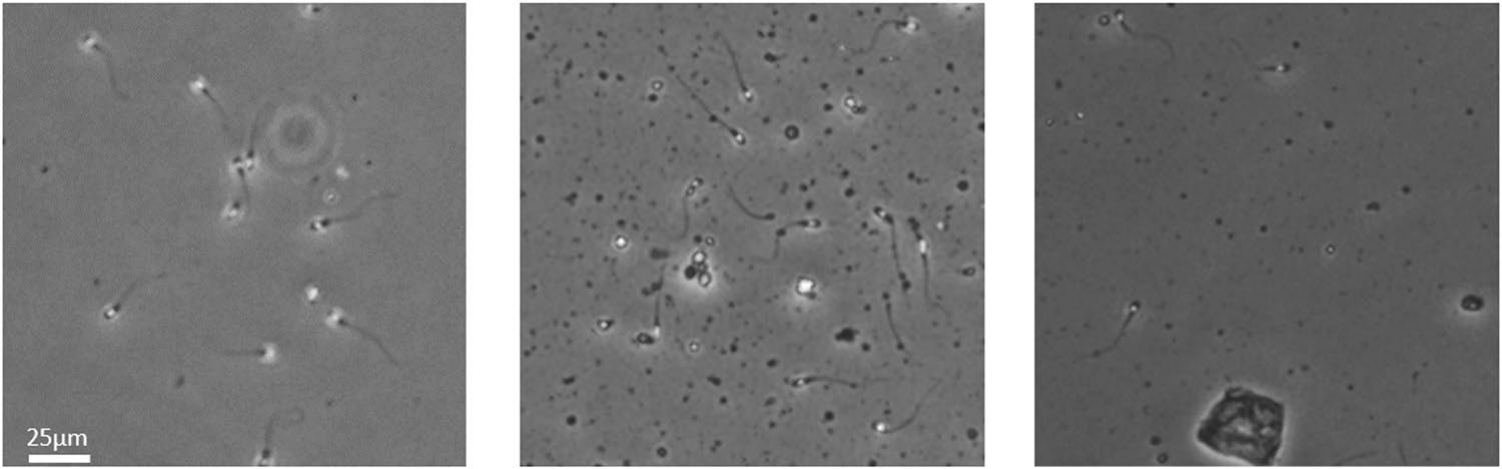
Three samples of recorded spermatozoa images: the brightness and focus of samples are different as well as their spermatozoa cell count and the presence of other irrelevant cells (like debris and blood cells), which should be considered during MTT. (Images are just a portion of a recorded frame and the full frame images are not presented in the figures for page alignment purposes).

The current study dataset was recorded in the Royan Institute Research Lab. A recording of the image sequences of human spermatozoa was conducted using the CASA software (Sperm Class Analyzer© Software Version 5.1; Microptics™). All samples were taken after obtaining informed consent from all subjects, or their legal guardians in accordance with relevant guidelines and regulations. The experimental protocol was approved by Royan Institute. The recording frequency was 50 consecutive digitized images per second (50 FPS) using a 10× negative phase-contrast objective (Ph1 DL). The analysis was performed using a chamber with a capacity for 10 µL and previously heated to 37 °C. The chamber was placed under the phase-contrast microscope (Nikon™ Eclipse E200) with a green filter and the images were captured using a video camera (Basler Vision Tecnologie A312FC). Two non-consecutive, randomly selected microscopic fields per sample were scanned. The captured image resolution was 768 by 576 pixels, and the colormap was 8-bit grayscale. The recorded samples were varied in terms of spermatozoa cell count, the existence of other cells (e.g., debris or blood cells), and noise level. Here, the spermatozoa cell count refers to the number of spermatozoa in the recording viewport or, more precisely, the number of tracks that exist during the recording time. Some of the recorded samples are shown in Fig. 1. Each pixel in the recorded images is 0.833 μm.

**Figure 2:**
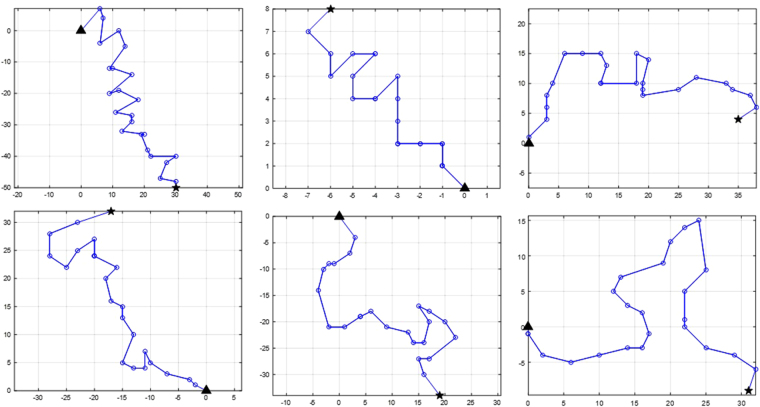
Some of the ground-truth tracks: the starting point is the filled triangle and the ending point is the filled star. The tracks are translated to the origin; so, the starting point of all the tracks is (0, 0). It should be noted that the velocities are different so the axis numbers might be taken into account for a better understanding of movements (the X and Y-axis units are in pixels).

After recording the data, some image sequences have been removed because of the unsettledness of the slide and cover-slip, or too much noise; 36 image sequences were selected as the final dataset of the current study. These image sequences were different in terms of cell count, brightness, noise condition, and the total motility of spermatozoa. There were at least four and at most 96 spermatozoa in the recorded image sequences, and the total number of spermatozoa was 1,659 in all the 36 image sequences; thus, the average spermatozoa cell count in an image sequence was about 46. This information has been summarized in Table 1. All the image sequences were of the same length (25 frames). For evaluating any MTT algorithm, the true track of each spermatozoon is required; thus, all of 1,659 spermatozoa tracks were precisely extracted manually by the Manual Tracking plugin of the FIJI software^44^. Track extraction was performed by a single well-trained operator under the supervision of an expert in the field. After the extraction, the dataset is ready for evaluating the MTT algorithm. Extracting tracks from captured image sequences provides a ground-truth and it could be confirmed that there are nonlinearities in spermatozoa movement. Some of the ground-truth tracks are depicted in Fig. 2, which shows this fact.

Flagellar beating is the main physiological cause of spermatozoon movement^45,46^. Spermatozoon tries to swim directly by means of sine-wavelike motions of the flagellum in order to reach the oocyte. However, the movement seems to have random fluctuations. There are many studies that investigated the movement of spermatozoon in 2D and 3D environments, and suggested complex curves and a formulation for its movement^46–50^. The complexity and nonlinearity suggest the usage of manually extracted tracks as a rich information source to model the movement of spermatozoa and the usage of the model to predict new tracks. More precisely, the problem involves calculating the probability $p({\tau }_{new}|{\mathscr{D}})$ instead of *p*(*τ*_*new*_), to achieve better results, where ${\mathscr{D}}$ is the manually extracted tracks dataset and *τ*_*new*_ is the new track that should be estimated from the observations. This approach is fully described in the following subsections.

### Observation basic definitions

In the MTT problem, there is a sequence of observations in a specific time interval 1, ..., *T* and the data association for each target should be performed using this sequence. In video and image processing cases, we have a set of acquired images; these are: *S*_*I*_ = {*I*_*t*_|*t* = 1, ..., *T*}. The observations are extracted from *S*_*I*_. Here, *t* is a discrete variable that indexes the time steps of sampling.

The observation step as an image-processing task is mainly an image segmentation that discriminates targets from the background or other non-target objects present in the current image. The output of a segmentation algorithm, performed on a single image, is a set of coordinates that represents the centroid of the detected targets that form the observation set for the current image:

1 $${o}_{t}\,=\,\{({\tilde{x}}_{t}^{i},{\tilde{y}}_{t}^{i})|i=1,\mathrm{...},{n}_{t}\}$$

In (1), *n*_*t*_ is the number of detected targets in the image *I*_*t*_. Let $O={\cup }_{t=1}^{T}{o}_{t}$ be the set of all the observations; then, the final output of the data association is the set of tracks and false alarms called *ω*. More precisely, *ω* = {*τ*_0_, *τ*_1_, *τ*_2_, …, *τ*_*K*_} in which *τ*_*i*_, *i* = 1, …, *K* are tracks with their associated observations, and *τ*_0_ is the set of all the unassigned observations or false alarms, and *K* is the number of all the detected targets or the number of tracks in the image sequence *S*_*I*_. From the definition of the data association, we have $O={\cup }_{i=1}^{K}{\tau }_{i}$, which means that the set of all observations is equal to the union of all the associated points in the tracks and false alarms. There are also some extra conditions for *τ*_*i*_, *i* = 1, …, *K* to ensure being the correct tracks:

2 $$\begin{array}{l}{\tau }_{i}\cap {\tau }_{j}=\varnothing \,\mathrm{for}\,i\ne j\\ |{t}_{i}|\,1\,{\rm{for}}\,i=1,\ldots ,K\\ |{\tau }_{i}\cap {o}_{t}|\le 1\,{\rm{for}}\,i=1,{\rm{\ldots }},\,K\,{\rm{and}}\,t=1,{\rm{\ldots }},T\end{array}$$

These conditions guarantee the uniqueness and the independence of all the tracks and also the fact that a track at least needs to be present in two frames of the observation sequence and at each frame the tracks should be allowed to have at most one observation assigned. A track may start in any frame *t* and terminate in any frame *t* + 1, ..., *T*.

### Phase-contrast properties for segmentation

**Figure 3:**
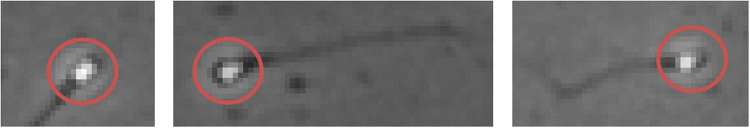
Images of spermatozoa head in phase-contrast microscopy images: there is extra brightness around the head in addition to the spermatozoa head itself (marked by red circles). The images are enlarged versions of the original recorded images to represent the related subject better.

**Figure 4:**
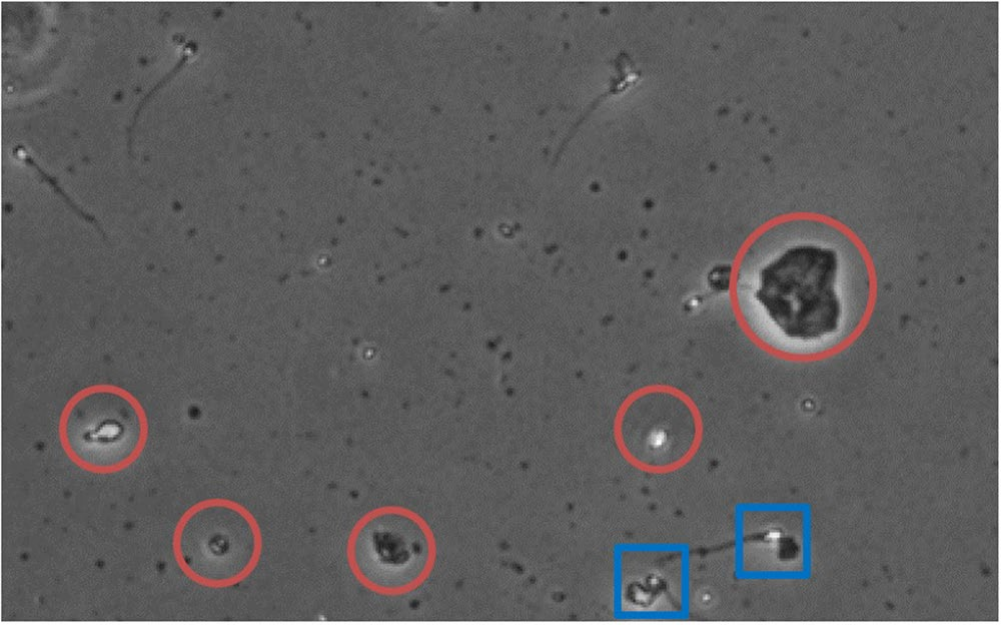
One sample (portion of a full recorded frame) that shows the extra brightness for the objects present in the image (marked with red circle), which makes discrimination and segmentation of spermatozoa (objects of interest) a difficult task to perform. The situation becomes more challenging when the spermatozoa are near an extra brightness and the white part of the objects partially or totally merge (marked with blue squares).

Phase-contrast microscopy creates artificial shadows as if there is a side illumination^51^. It helps to make better contrast, and therefore, provides an improved view of the detail structures of the transparent specimen. However, the contrast enhancement has side effects such as producing extra brightness around the objects (Fig. 3). Additional illumination could prevent the correct segmentation of objects of interest from the background and other objects present in the image. Figure 4 shows certain ambiguities that makes segmentation and observation difficult.

### Evaluation of observation

There are certain definitions and quantities for evaluating the observation algorithm; these include the probability of detecting targets (*p*_*d*_), the probability of missing targets (*p*_*m*_), and the rate of detecting non-targets as targets called false alarm rate (*FAR*). It is obvious that we have

3 $${p}_{d}+{p}_{m}=1$$

These probabilities are the properties of an observation algorithm. Now, we describe the relations for calculating these quantities in our dataset. After segmentation, we would have a set of coordinates as the results. We should evaluate these coordinates by comparing them with the ground-truth coordinates and finally calculating *p*_*d*_, *p*_*m*_, and *FAR*. If we have ${o}_{t}=\{({\tilde{x}}_{t}^{i},{\tilde{y}}_{t}^{i})|i=1,\mathrm{...},{n}_{t}\}$ as the segmentation output and ${g}_{t}=\{({x}_{t}^{j},{y}_{t}^{j})|j=1,\mathrm{...},{m}_{t}\}$ as the ground-truth, for time step *t*, we can calculate the detection probability and false alarm rate from these two sets. The number of truly detected objects from *o*_*t*_ that match (within a 5 pixel or 4.17 μm radius) with corresponding objects in *g*_*t*_ is related to the detection probability. If we assume ${n}_{t}^{TP}$ as the number of truly detected objects (True Positive) from *o*_*t*_, then, the detection probability in the current time step would be

4 $${p}_{d,t}=\frac{{n}_{t}^{TP}}{{m}_{t}}$$

For a complete image sequence *S*_*I*_, we can calculate ${\bar{p}}_{d}$ as the mean of *p*_*d*,*t*_ over different values of *t* (different frames) as follows:

5 $${\bar{p}}_{d}=\frac{{\sum }_{t=1}^{T}{p}_{d,t}}{T}$$

For the overall calculation of *p*_*d*_ in a series of image sequences (a whole dataset), we can average the overall ${\bar{p}}_{d}$ as follows:

6 $${p}_{d}=\frac{{\sum }_{{S}_{I}\in {\mathscr{S}}}{\bar{p}}_{d}({S}_{I})}{\Vert {\mathscr{S}}\Vert }$$

In (6), $\Vert {\mathscr{S}}\Vert$ is the cardinal of the dataset, i.e., the number of image sequences (*S*_*I*_) in the ${\mathscr{S}}$, and ${\bar{p}}_{d}({S}_{I})$ is the average detection probability of *S*_*I*_. We can now calculate the probability of missing an object as follows: *p*_*m*_ = 1 − *p*_*d*_.

**Figure 5:**
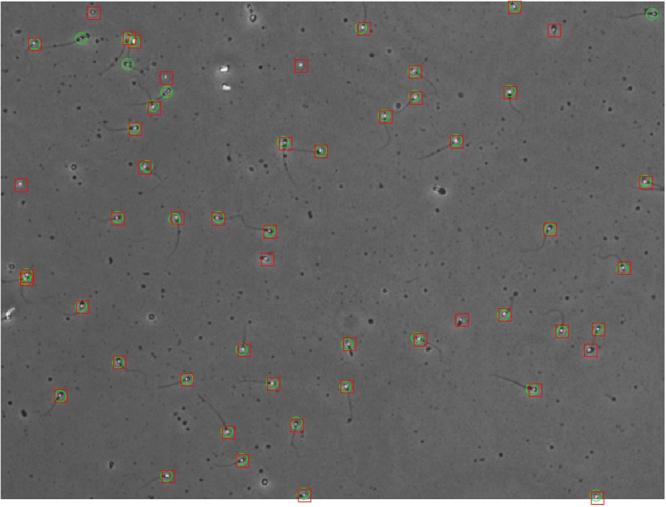
Two sets of ${o}_{t}=\{({\tilde{x}}_{t}^{i},{\tilde{y}}_{t}^{i})|i=1,\mathrm{...},{n}_{t}\}$ as the segmentation output and ${g}_{t}=\{({x}_{t}^{j},{y}_{t}^{j})|j=1,\mathrm{...},{m}_{t}\}$ as the ground-truth depicted on a sample frame of an image sequence. Green circles are elements of *g*_*t*_ and red squares are elements of *o*_*t*_. As observable, there are eight false alarms (red squares without green circles) and four missed detections (green circles without red squares).

Similarly, the *FAR* in a single frame is ${n}_{t}-{n}_{t}^{TP}$. The only problem that remains here is the way of matching the points in *o*_*t*_ with those in *g*_*t*_. The matching problem is a very common problem in MTT, both for the location of the objects in a frame and as well as for matching the final tracks with the ground-truth. For solving this matching problem (which is originally NP-Hard), many MTT studies like^52^ have used a standard polynomial method called the Hungarian or the Munkres algorithm^53^. Building the mutual Euclidean distance matrix for the elements of *o*_*t*_ and *g*_*t*_, and using the Munkres algorithm, we can match the points in two sets. It should be mentioned that distances more than 5 pixels were defined as unacceptable (infinite distance in the distance matrix entry). That is because the head of a normal spermatozoon is an ellipse with average dimensions of 4.3 μm by 2.9 μm^54^, which means 5.2 pixel by 3.5 pixel in our images (0.833 μm/pixel). We take the ellipse major axis length, which is 5 pixels, as the maximum acceptable distance for assuming two objects as a matched pair in the two sets. Figure 5 shows two sets of *o*_*t*_ and *g*_*t*_ in a sample frame.

### Segmentation algorithm

In this research, the observation step has been implemented in four steps in each frame of an image sequence:

1. Converting image to binary (black and white) by adaptive image threshold using local first-order statistics^55^
2. Applying closing morphological operation^56^ on the resulted binary image with a circular structuring element with radius *r*_*SE*_ pixels
3. Filling the holes of the segmented objects
4. Filtering segmented objects, that is, keeping objects with a blob area between *β*_*min*_ and *β*_*max*_

Setting a threshold for turning the image into binary is very important because the resultant binary image is the basis for the following steps. We have used the adaptive image threshold using local first-order statistics^55^ for each frame’s segmentation. As there might be several particles other than spermatozoa are segmented as foreground, for achieving a better result, certain additional processes on the resulted image are necessary.

Steps 2 and 3 of the segmentation algorithm are conducted for connecting parted big-components that are not related to the spermatozoa. If a big component is being parted into a few smaller components, they may be classified in Step 4 as a spermatozoon head; thus, connecting the parted components and filling their holes, which is necessary to avoid many more false positives. *r*_*SE*_ in Step 2 was set to 5 pixels after sweeping that parameter from 2 to 10 pixels for getting the best performance (high *p*_*D*_ and low *FAR*).

**Figure 6:**
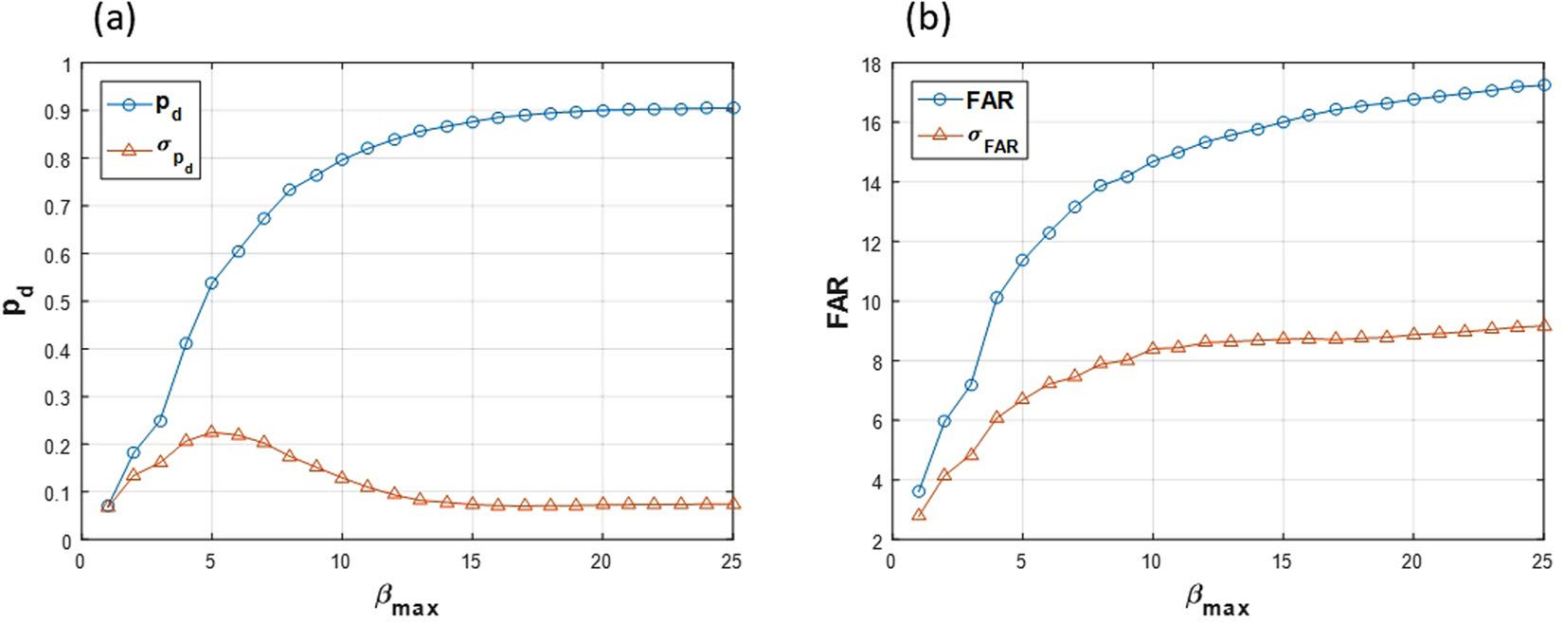
*p*_*d*_ and *FAR* plotted vs. *β*_*max*_ (**a**) Average detection probability (*p*_*d*_) and its standard deviation among all the image sequences of the dataset is plotted vs. *β*_*max*_ (**b**) Average false alarm rate (*FAR*) and its standard deviation among all the image sequences of the dataset is plotted vs. *β*_*max*_.

In Step 4, *β*_*min*_ is set to 1 pixel because there are always spermatozoa heads that had as little as 1 pixel area after steps 1–3. Increasing *β*_*min*_ to even two pixels and setting it to 3 causes the maximum *p*_*D*_ to decrease to about 10%. Sweeping *β*_*max*_ from 1 to 25, we achieve a broad range of *p*_*D*_ and *FAR* (Fig. 6). The area of normal spermatozoa is in the 8.5.0.12.2 μm^2^ interval^54^, which means 12.18 pixels; thus, there is no need for sweeping *β*_*max*_ by more than 25 pixels for filtering the heads of spermatozoa. Figure 6 shows the resulting *p*_*D*_ and *FAR* after sweeping *β*_*max*_ from 1 to 25 pixels. Thus, we might have different values of *p*_*D*_ by setting *β*_*max*_ to different values.

The final segmentation accuracy could be enhanced by designing more sophisticated segmentation methods, which can be the objective of different independent studies like^57–59^.

### Post-segmentation processing

**Figure 7:**
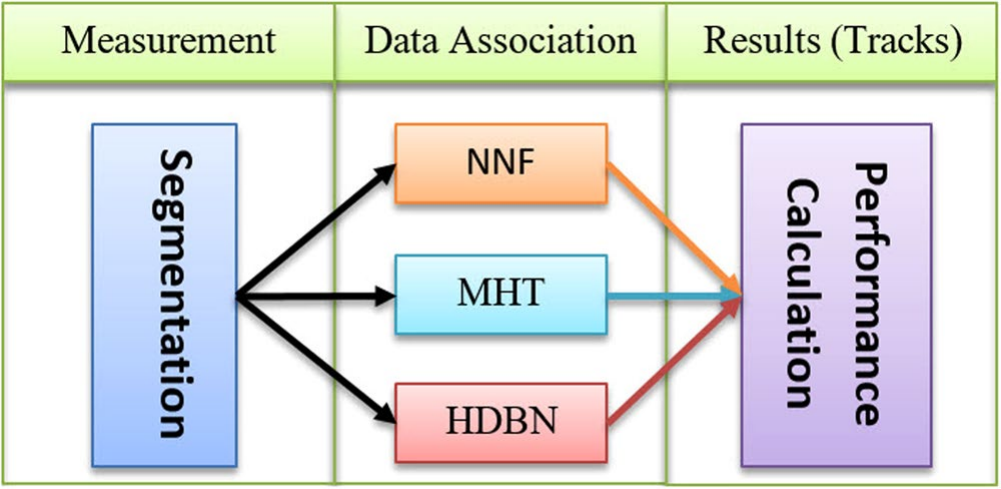
Schematic design for comparing the current study results with other well-known algorithms (MHT and NNF): the observation part is common across the three methods, but the data association is different in each method. In the last part, the performance of each algorithm will be calculated.

After observation, data association should be performed. All MTT algorithms need observations in each time step (frame) as input. This input is very important for the algorithm because if the observation is not so accurate, then, the data association results would also be erroneous. In this study, we have prepared multiple observation qualities and then input these qualities into different well-known algorithms and as well as our algorithm. After that, we could compare the different algorithms. The algorithms shared the same observation but had a different data association algorithm (Fig. 7). The next subsection completely describes our approach and method for data association.

### Hybrid network definition

Probabilistic Graphical Models (PGM) have been developed for modeling the relationship between random variables and for inference based on partial observations. As noted in “Introduction” Section, DBNs are used to handle the uncertainty of a system evolution over time. Typical DBNs have discrete random variables, and therefore, their CPD is often represented as tables (sometimes called Conditional Probability Table or CPT). The final goal of building a BN or a DBN is to represent the full joint distribution of all the random variables in the network. Assuming that there are *n* variables {*X*_1_, *X*_2_, …, *X*_*n*_}, the full joint distribution can be expressed using the chain rule of the BNs:

7 $$p({X}_{1},{X}_{2},\ldots ,{X}_{n})=\prod _{i=1}^{n}p({X}_{i}|Pa({X}_{i}))$$

In the above equation, *Pa*(*X*_*i*_) is the set of nodes which are the parents of *X*_*i*_ and each *p*(*X*_*i*_|*Pa*(*X*_*i*_)) is a CPD.

BNs and DBNs can also include continuous variables besides discrete variables, which are called hybrid networks. In the case of a discrete child with continuous parents, assuming that continuous parents are ***Z*** = {*Z*_1_, *Z*_2_, …, *Z*_*N*_} and the discrete child is *U* which has *m* possible values {*u*_1_, *u*_2_, …, *u*_*m*_}, the CPD for *U* is defined as follows (as mentioned in^34^):

8 $$\begin{array}{rcl}p(U={u}_{j}|{\boldsymbol{Z}}) & = & \sum _{r=1}^{R}{w}^{r}{p}_{j}^{r}\\ {w}^{r} & = & \frac{\exp ({\zeta }_{0}^{r}+{\sum }_{i=1}^{N}{\zeta }_{i}^{r}{Z}_{i})}{{\sum }_{q=1}^{R}\exp ({\zeta }_{0}^{q}+{\sum }_{i=1}^{N}{\zeta }_{i}^{q}{Z}_{i})}\end{array}$$

**Figure 8:**
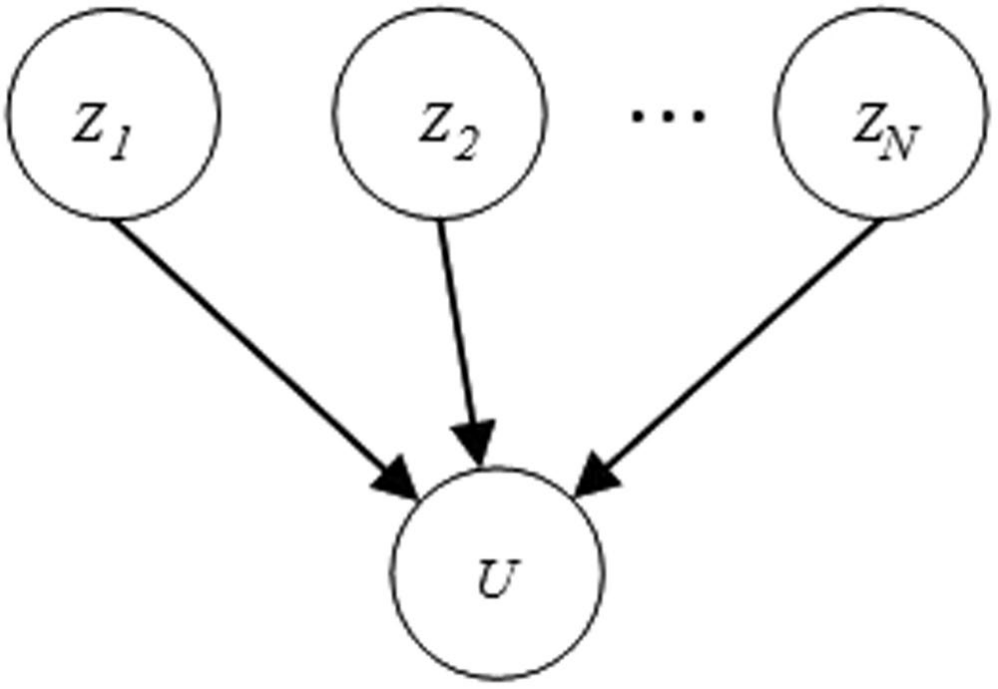
Graphical model of continuous parents {*Z*_1_, *Z*_2_, …, *Z*_*N*_} with a discrete child *U*.

In (8), ${p}_{j}^{r}$ are the probability values over *u*_1_, *u*_2_, …, *u*_*m*_ for the region *r*(1 ≤ *r* ≤ *R*), which means

9 $$\sum _{j=1}^{m}{p}_{j}^{r}=1$$

and ${\zeta }^{r}={[{\zeta }_{0}^{r},{\zeta }_{1}^{r},\ldots ,{\zeta }_{N}^{r}]}^{T}$ is a vector of weights for the region *r* (the space has been partitioned into *R* regions, in which *R* has been arbitrarily chosen based on the model). We have designed our model based on the Softmax-CPD by partitioning the space of all possible tracks into *N* parts and calculating the probability for each candidate point based upon each region. The graphical representation is depicted in Fig. 8. This formulation describes the conditional probability distribution for choosing between the discrete values of *u*_*i*_.

This part has described the HDBN formulation in general; from now on, our method to adapt HDBN for solving the MTT problem is described.

### Track normalization

One of the goals of the current study for building a graphical model for data association involves using the existing data of the manually extracted tracks, i.e., calculating $p({\tau }_{new}|{\mathscr{D}})$ instead of just *p*(*τ*_*new*_), where ${\mathscr{D}}$ is manually extracted tracks dataset and *τ*_*new*_ is the new track which should be estimated from the observations. More precisely, there are a set of manually extracted tracks like the ones in Fig. 2; these tracks can be used as a basis for comparison and selecting the best observation for a new track being tracked. There are several ways for training a supervised system based on the aforementioned data, but the preparation of the data is more important here, i.e., what is the feature vector for the similarity observation between the tracks and how can it help to solve the data association section of MTT.

A track is a set of points in two-dimensional space, i.e., $\tau =\{({x}_{{t}_{1}},{y}_{{t}_{1}}),\ldots ,({x}_{{t}_{1}+L-1},{y}_{{t}_{1}+L-1})\}$, where *t*_1_ is the start point and *L* is the length of the track sequence. Now for finding similar *patterns* of movement in ${\mathscr{D}}$, there must be a *normalization* in the tracks, which removes the initial direction variations, so the tracks could be compared. For normalizing a track, first it must be represented in a polar way: a track can be redefined as $\tau =\{({d}_{{t}_{1}},{\theta }_{{t}_{1}}),\ldots ,({d}_{{t}_{1}+L-1},{\theta }_{{t}_{1}+L-1})\}$, where *d*_*i*_ is the displacement from point *i* to point *i* + 1, and *θ*_*i*_ is its relevant angle with respect to the X-axis:

10 $$\begin{array}{rcl}{d}_{i} & = & \sqrt{{({x}_{i+1}-{x}_{i})}^{2}+{({y}_{i+1}-{y}_{i})}^{2}}\\ {\theta }_{i} & = & \arctan (\frac{{y}_{i+1}-{y}_{i}}{{x}_{i+1}-{x}_{i}})\end{array}$$

**Figure 9:**
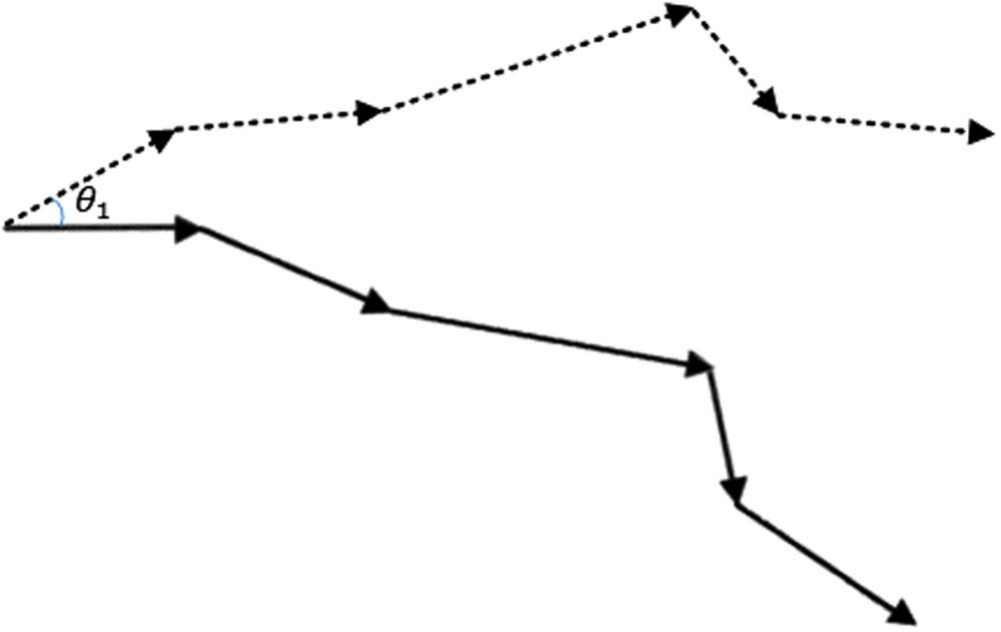
The original track (dotted) and the normalized track (solid); the normalized track does not have an initial angle *θ*_1_ like the original track and has a zero angle in its first move.

Now, if the track is rotated with the angle $-{\theta }_{{t}_{1}}$, it is normalized; so, the first displacement is always exactly in the horizontal direction (zero degrees with respect to the X-axis), and it can be compared to the other tracks while the initial direction is removed (Fig. 9). After normalization, every track has zero degrees in the first element: $\tau =\{({d}_{{t}_{1}},0),\ldots ,({d}_{{t}_{1}+L-2},{\theta }_{{t}_{1}+L-2})\}$. It should be noted that if a cell is immotile, then the related angle of movement in that step was set to zero.

### Design HDBN for data association

In the data association for an image sequence *S*_*I*_, the input is $O={\cup }_{t=0}^{T}{o}_{t}$ and the output is *ω* = {*τ*_0_, *τ*_1_, *τ*_2_, …, *τ*_*K*_}. For using the manually extracted tracks dataset (${\mathscr{D}}$ which consists of all manually extracted tracks for all image sequences) as a source of information for a specific image sequence *S*_*I*_, first, all of the manually extracted tracks of *S*_*I*_ are removed from ${\mathscr{D}}$ and the rest of manually extracted tracks are used; so, for each of the image sequences, its related data is pulled out because of the validity of the final results acquired (not using the image sequence manually extracted data which is currently being tracked). This is a standard method in cross validation called Leave-One-Out Cross Validation (LOOCV)^60^. Assuming that there are *N* manually extracted tracks left in ${\mathscr{D}}$ as a reference for comparison (${\tau }_{1}^{{\mathscr{D}}},{\tau }_{2}^{{\mathscr{D}}},{\rm{\ldots }},{\tau }_{N}^{{\mathscr{D}}}$), In the following we describe how to use these tracks as an information source for the data association of a new track. A new track is built step by step by assigning a new observation from the set of all observations. If we call a new track *i* until time *t*, ${{\rm{\Gamma }}}_{t}^{i}=\{({d}_{1}^{i},{\theta }_{1}^{i}),\ldots ,({d}_{t}^{i},{\theta }_{t}^{i})\}$, which will progress to the next time to build ${{\rm{\Gamma }}}_{t+1}^{i}$, in the end, it will be the *i*th track *τ*_*i*_, i.e. ${{\rm{\Gamma }}}_{T(i)}^{i}={\tau }_{i}$, in which *T*(*i*) is the length of the track *τ*_*i*_. Now, the partial likelihood of ${{\rm{\Gamma }}}_{t}^{i}$ and a track in ${\mathscr{D}}$ can be calculated using the inverse of *Z*_*t*,*j*_, and the distance between ${{\rm{\Gamma }}}_{t}^{i}$ and ${\tau }_{j}^{{\mathscr{D}}}(t)$ with the following definitions:

11 $$\begin{array}{rcl}\mathop{{Z}_{t,j}}\limits_{j=1\cdots N} & = & dist\,({{\rm{\Gamma }}}_{t}^{i},{\tau }_{j}^{{\mathscr{D}}}(t))=\sqrt{\sum _{t^{\prime} =1}^{t}{({d}_{t^{\prime} }^{i}-{d}_{t^{\prime} }^{j})}^{2}+{({\theta }_{{t}^{\text{'}}}^{i}-{\theta }_{t^{\prime} }^{j})}^{2}}\\ {\tau }_{j}^{{\mathscr{D}}}(t) & = & \{({d}_{1}^{j},{\theta }_{1}^{j}),\ldots ,({d}_{t}^{j},{\theta }_{t}^{j})\}\subseteq {\tau }_{j}^{{\mathscr{D}}}\end{array}$$

**Figure 10:**
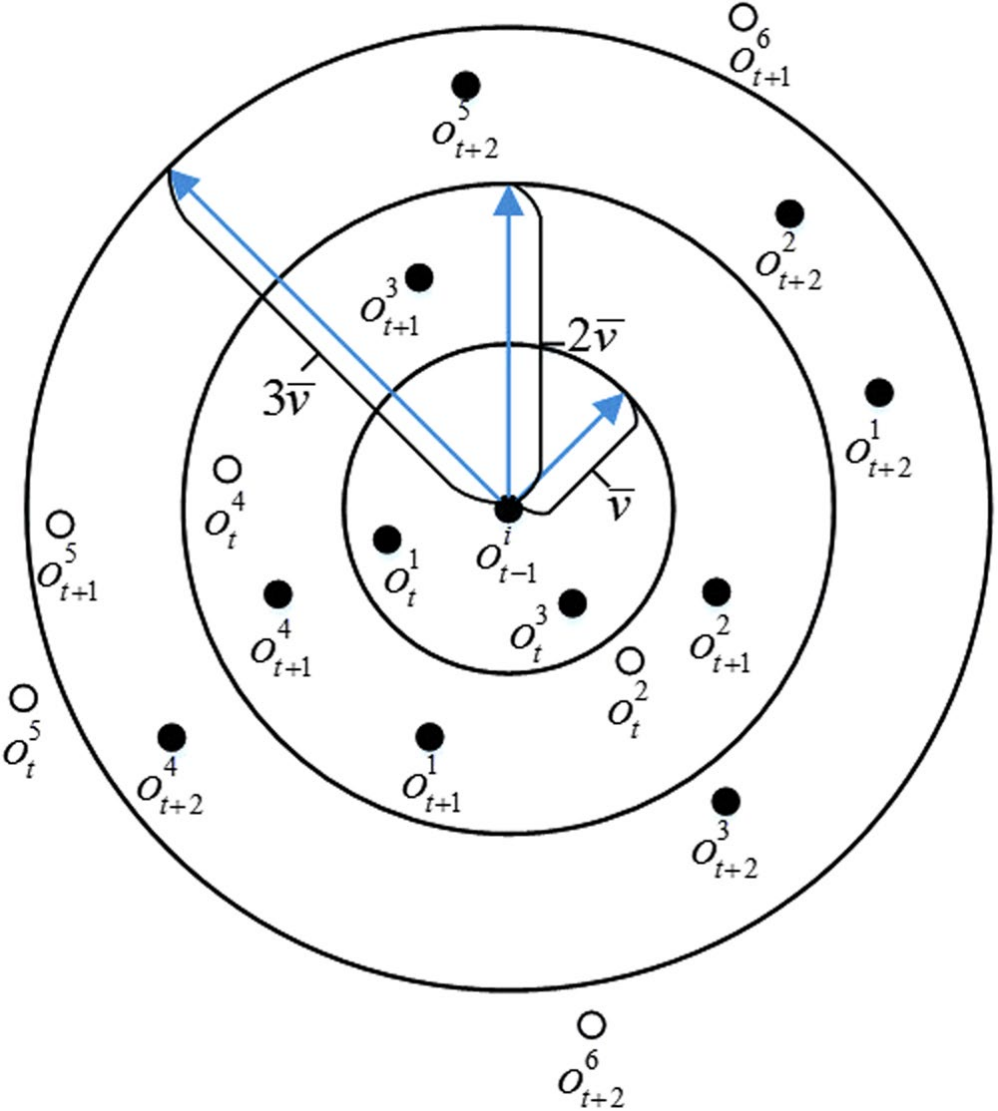
Candidates of the following points of track *τ*_*i*_ at time *t* − 1 from the set of related observations in the neighborhood circles, up to three consecutive missing observations: ${o}_{t}^{j}$ is the *j*th observation in the time step *t*. If ${o}_{t}^{j}$ is within the range of gated observations with respect to the last point (${o}_{t-1}^{i}$), then it is a possible candidate for being the next point on the track. If it is out of the range, however, it is an impossible candidate in time step *t*. The filled circles are possible candidates and the empty circles are impossible candidates at each time step.

In (11), *d*’s are in units of pixels and θ’s are in units of radians. In each step for building ${{\rm{\Gamma }}}_{t+1}^{i}$ from ${{\rm{\Gamma }}}_{t}^{i}$, there may be a missing observation in the track (which is marked by ${0}_{t}^{i}$). This will occur if there is no proper match for the current track *i* at time *t*, either because of an error in the observation system or due to the occlusion of the target in the current frame. The maximum number of consecutive missing observations of any track must be less than or equal to a specific threshold called $\bar{d}$ and; if this threshold is passed, the track should be terminated. There will be a neighborhood circle for each point in a track based on the maximum directional speed of the targets in all the image sequences ($\bar{v}$) such that the candidates of the next point of the track must be inside that circle. These two facts are depicted in Fig. 10, in which an end point in Track *τ*_*i*_ is the center of the figure (${o}_{t-1}^{i}$) and the possible candidates for the next Step *t* are in a circle with the radius equal to the magnitude of $\bar{v}$. Note that the observations in time *t* that are farther than $\bar{v}$ are marked as impossible (empty circles). In the case of the missing observation in time *t*, the following possible candidates at time *t* + 1 must be in the $2\bar{v}$ radius of the end point and so on for the next missing observations till the $\bar{d}$ threshold, which is three in Fig. 10. It should be noted that in the calculation of *Z*_*t*,*j*_ in (11), if at any *t*^'^ a track point was missing between 1 to *t*, a dummy point was considered with an equal distance between its previous and following observations. This should be performed so that the distance calculation becomes feasible.

**Figure 11:**
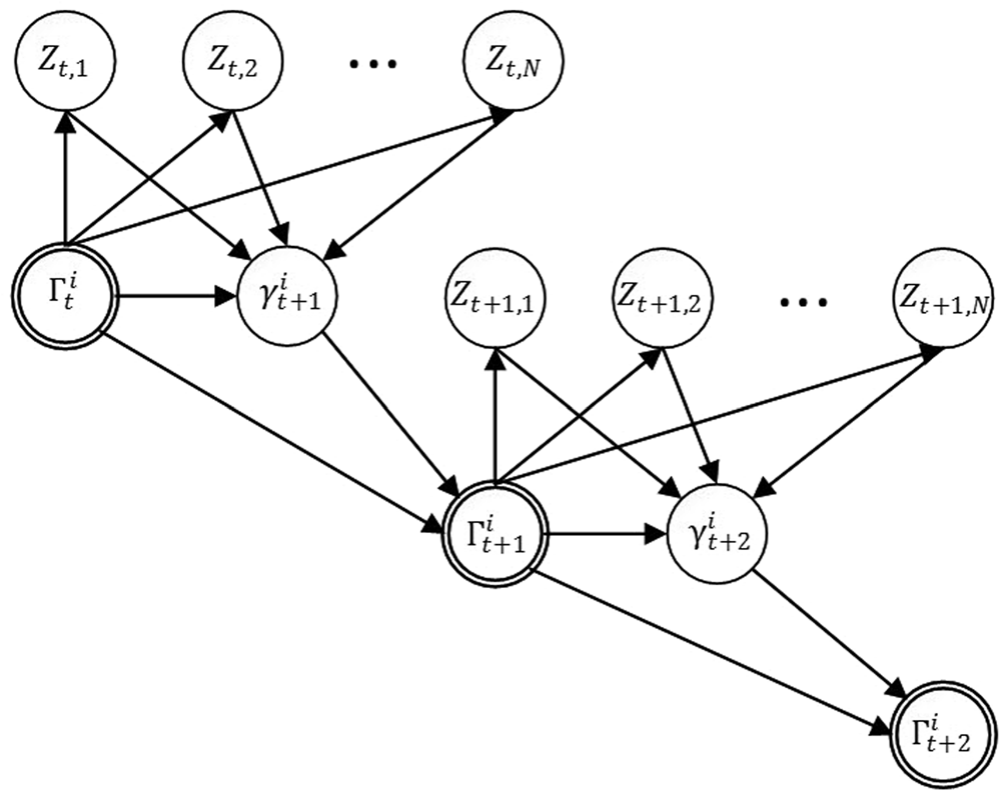
The HDBN model for data association of the track *τ*_*i*_: *Z*_*t*,*j*_ (which is the continuous node in the HDBN) is the distance between the *i*th track (*τ*_*i*_) the *j*th track in the dataset up to time t. ${\gamma }_{t+1}^{i}$ is a discrete node; it is the next point (observation) which must be assigned to track *τ*_*i*_. It has *m* states (based on the gated observations set ${N}_{t}^{i}$) with a specific probability for each of them. The next point of the track is selected based on the probability values $p({\gamma }_{t+1}^{i}={u}_{j}|{{\boldsymbol{Z}}}_{t})$, in which *u*_*j*_ is a point from the candidate points in the gated neighborhood set and ***Z***_***t***_ = {*Z*_*t*,1_, *Z*_*t*,2_, …, *Z*_*t*,*N*_}. ${\Gamma }_{t}^{i}$ is the track *τ*_*i*_ completed up to time *t* and will be used for calculating *Z*_*t*,*j*_, and finally, ${\gamma }_{t+1}^{i}$.

Now, based on the above definitions and descriptions, the suggested HDBN model for data association in the MTT problem is as depicted in Fig. 11. The continuous nodes in the HDBN model are *Z*_*t*,*j*_(*j* = 1 … *N*) and the discrete node is ${\gamma }_{t}^{i}$ (next observation of the *i*th track in time index *t*) which has $m=\Vert {N}_{t}^{i}\Vert$ states; thus, we have

12 $${N}_{t}^{i}=\{({\tilde{x}}_{t}^{j},{\tilde{y}}_{t}^{j})|d(({\tilde{x}}_{t}^{j},{\tilde{y}}_{t}^{j}),({x}_{{t}_{l}}^{i},{y}_{{t}_{l}}^{i})) < (t-{t}_{l})\bar{v}\}$$

In (12), ${N}_{t}^{i}\subseteq {o}_{t}$ and it contains some candidate points for the selection of the next point which is in the neighborhood circle. *d*((*x*_1_, *y*_1_), (*x*_2_, *y*_2_)) denotes the Euclidean distance between two points, *t*_*l*_ is the last time index in which the track had an assigned observation, and we have

13 $$t-\bar{d}\le {t}_{l}\le t-1$$

Different states of ${\gamma }_{t}^{i}$ can be obtained from the members of ${N}_{t}^{i}$ in (12) as follows:

14 $${\gamma }_{t}^{i}\in \{{u}_{j}=({d}_{t}^{j},{\theta }_{t}^{j})|1\le j\le m\}$$

The states of ${\gamma }_{t}^{i}$ are obtained from the possible candidate points in the neighborhood circle (members of ${N}_{t}^{i}$), which are converted to polar representation. Based on the relations of HDBN, the probability of selecting any point in the neighborhood circle is as follows:

15 $$p({\gamma }_{t}^{i}={u}_{j}|{{\boldsymbol{Z}}}_{t})=\sum _{r=1}^{N}{w}_{t}^{r}{p}_{j}^{r}(t)$$

16 $${w}_{t}^{r}=\frac{\exp ({\zeta }_{0}^{r}+{\sum }_{i=1}^{N}{\zeta }_{i}^{r}{Z}_{t,i})}{{\sum }_{q=1}^{N}\exp ({\zeta }_{0}^{q}+{\sum }_{i=1}^{N}{\zeta }_{i}^{q}{Z}_{t,i})}$$

Here, the selected coefficient is ${\zeta }_{i}^{q}=-\delta (i-q)$ and *δ* is the Kronecker delta function which will result in

17 $${w}_{t}^{r}=\frac{\exp (-dist({{\rm{\Gamma }}}_{t}^{i},{\tau }_{r}^{{\mathscr{D}}}(t)))}{{\sum }_{q=1}^{N}\exp (-dist({{\rm{\Gamma }}}_{t}^{i},{\tau }_{q}^{{\mathscr{D}}}(t)))}$$

The *R* regions introduced earlier is just the number of regions and it could be chosen arbitrarily in the model^34^. We designed our model based on the Softmax-CPD by partitioning the space of all possible tracks into *N* parts (*R* = *N*) and calculating the probability for each candidate point based upon each region. This will result in higher weights as a result of greater similarity between the current track and the tracks in ${\mathscr{D}}$ and lower weights for less partial likelihood between the current track and the tracks in ${\mathscr{D}}$. For probability distribution over the possible values of ${\gamma }_{t}^{i}$, a Gaussian distribution is defined as follows:

18 $$\begin{array}{rcl}{\tilde{p}}_{j}^{r}(t) & = & p(({d}_{j},{\theta }_{j})|({D}_{t}^{r},{\Theta }_{t}^{r})) \sim {\mathscr{N}}({[{d}_{j},{\theta }_{j}]}^{T};{\mu }_{t}^{r},{{\rm{\Sigma }}}_{t}^{r})\\ {D}_{t}^{r} & = & {d}_{1:t}^{r}\\ {\Theta }_{t}^{r} & = & {\theta }_{1:t}^{r}\end{array}$$

**Figure 12:**
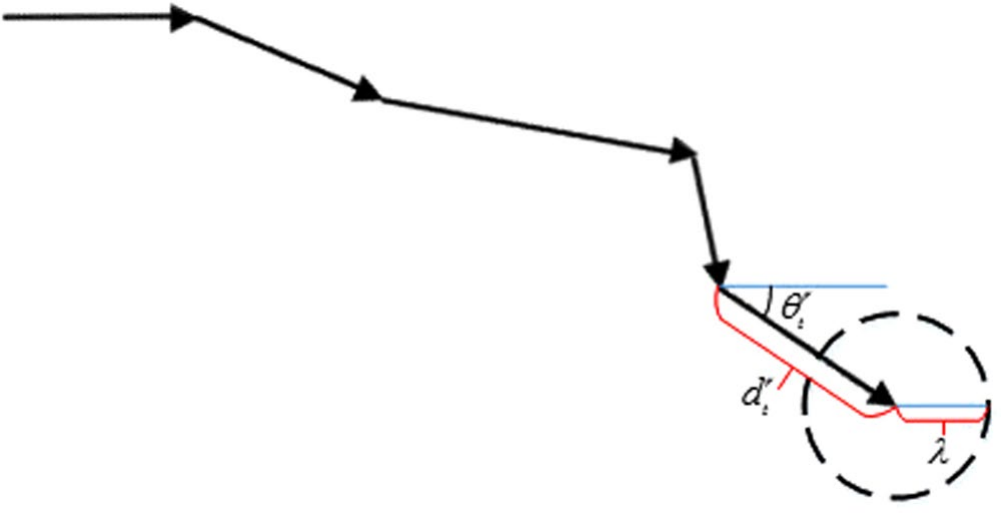
Mean and covariance parameters in ${\tilde{p}}_{j}^{r}(t)$.

The normal distribution of the angle can be interpreted by generalizing the concept of angle from *θ* to *2kπ* + *θ*, or by mapping ${\mathbb{R}}$ to unit circle which is known as wrapped normal distribution^61^. The mean of this Gaussian distribution is the current point of the track in ${\mathscr{D}}$, i.e., ${\mu }_{t}^{r}={[{d}_{t}^{r},{\theta }_{t}^{r}]}^{T}$, and the covariance matrix is a function of the current distance, which means the higher the current distance is, the bigger the covariance matrix determinant will be (Fig. 12):

19 $${{\rm{\Sigma }}}_{t}^{r}=\lambda {d}_{t}^{r}{I}_{2\times 2}$$

*λ* is a coefficient determining the broadness of the distribution over ${\mu }_{t}^{r}$.

The goal of this step is to score all of the *m* points in ${N}_{t}^{i}$ in *τ*_*j*_’s point of view for all ${\tau }_{j}\in {\mathscr{D}}$. This score is then multiplied by ${w}_{t}^{r}$ as in (15), the partial likelihood between *τ*_*i*_ and *τ*_*j*_ in time step *t*. The Gaussian distribution is used as a natural choice for a symmetric and decreasing-from-the-center function because as the point goes away from predefined dataset track point, its probability (likelihood to be in the pattern of the current dataset track) should be decreased. It should be mentioned that other 2D symmetric probability distributions could be used instead of Gaussian, like a conical shape or other possible distributions that are symmetric and decrease from the center point to the sides.

According to Equation (9), we must have a probability distribution, so, the normalization constant is required in the Equation (18):

20 $$\begin{array}{rcl}{\sigma }^{r}(t) & = & \sum _{j=1}^{m}{\tilde{p}}_{j}^{r}(t)\\ {p}_{j}^{r}(t) & = & \frac{{\tilde{p}}_{j}^{r}(t)}{{\sigma }^{r}(t)}\end{array}$$

Here, the Equation (15) definition is complete. Now, the next point in a track must be selected. The next point is the point with the maximum probability among all the candidate points:

21 $$\widehat{{\gamma }_{t+1}^{i}}={\rm{\arg }}\,\mathop{\max }\limits_{{\gamma }_{t+1}^{i}}\,p({\gamma }_{t+1}^{i}|{{\boldsymbol{Z}}}_{t})$$

In addition, this method gives promising results (close to the optimal solution); it is a suboptimal solution because there are multiple tracks at time *t* that must be assigned a new point (observation); so, this is a multiple assignment optimization problem. The multiple assignment problem is originally NP-Hard but it could be solved in polynomial time using the Munkres algorithm^53^ like the matching problem mentioned in “Evaluation of observation” Section. Using this algorithm, in each step, the best candidates for completing all the tracks up to this stage are chosen based on the probability distribution $\,p({\gamma }_{t+1}^{i}|{{\boldsymbol{Z}}}_{t})$ for each track *τ*_*i*_. It is well worth mentioning that there are two different approaches for solving MTT problems: single scan and multi scan^26^. The approach of the current study is single scan which have been implemented and reported. The HDBN MTT algorithm is summarized in Table 2.

**Table 2:** Hdbn Mtt Algorithm.

**Algorithm 1**
**Initializations:** ${\boldsymbol{\omega }}\,=\,\varnothing$ Remove ***S***_***I***_ data from ${\mathscr{D}}$ and prepare ${{\boldsymbol{\tau }}}_{1}^{{\mathscr{D}}},{{\boldsymbol{\tau }}}_{2}^{{\mathscr{D}}},{\rm{\ldots }},{{\boldsymbol{\tau }}}_{{\boldsymbol{N}}}^{{\mathscr{D}}}$ **For** ***t*** ** = 1 to** ***T*** Segment ***I***_***t***_ to obtain ***O***_***t***_ **For** ***i* = 1 to** ***K*** **Form** ${{\boldsymbol{N}}}_{{\boldsymbol{t}}}^{{\boldsymbol{i}}}$ **for** ${{\boldsymbol{\tau }}}_{{\boldsymbol{i}}}$ **based on** $\bar{{\boldsymbol{v}}}$ **and** $\bar{{\boldsymbol{d}}}$ ${\boldsymbol{p}}({{\boldsymbol{\gamma }}}_{{\boldsymbol{t}}+1}^{{\boldsymbol{i}}}|{{\boldsymbol{Z}}}_{{\boldsymbol{t}}})\,=\,\sum _{{\boldsymbol{r}}=1}^{{\boldsymbol{N}}}\frac{{\exp }(-{\boldsymbol{dist}}({{\boldsymbol{\Gamma }}}_{{\boldsymbol{t}}}^{{\boldsymbol{i}}},{{\boldsymbol{\tau }}}_{{\boldsymbol{r}}}^{{\mathscr{D}}}({\boldsymbol{t}})))}{{\sum }_{{\boldsymbol{q}}=1}^{{\boldsymbol{N}}}{\exp }(-{\boldsymbol{dist}}({{\boldsymbol{\Gamma }}}_{{\boldsymbol{t}}}^{{\boldsymbol{i}}},{{\boldsymbol{\tau }}}_{{\boldsymbol{q}}}^{{\mathscr{D}}}({\boldsymbol{t}})))}{{\boldsymbol{p}}}_{{\boldsymbol{j}}}^{{\boldsymbol{r}}}({\boldsymbol{t}})$ **End For** Assign ${{\boldsymbol{\gamma }}}_{{\boldsymbol{t}}+1}^{i\ast }$ for each track ${{\boldsymbol{\tau }}}_{{\boldsymbol{i}}}$ by Munkres algorithm **For** ***i*** = 1 to *K* **If** ${{\boldsymbol{\gamma }}}_{{\boldsymbol{t}}+{\bf{1}}}^{i\ast }={{\bf{0}}}_{{\boldsymbol{t}}+{\bf{1}}}^{{\boldsymbol{i}}}$ ${{\boldsymbol{m}}}_{{\boldsymbol{t}}+1}^{{\boldsymbol{i}}}$**+ +** **If** ${{\boldsymbol{m}}}_{{\boldsymbol{t}}+1}^{{\boldsymbol{i}}}\ge \bar{{\boldsymbol{d}}}$ Terminate ${{\boldsymbol{\tau }}}_{{\boldsymbol{i}}}$ ***K−−*** **End If** **Else** ${{\boldsymbol{m}}}_{{\boldsymbol{t}}+1}^{{\boldsymbol{i}}}$**=0** **End If** **End For** Update ${\boldsymbol{\omega }}$ based on ${{\boldsymbol{\tau }}}_{{\boldsymbol{i}}},1\le {\boldsymbol{i}}\le {\boldsymbol{K}}$ **End For**

## Results and Discussion

The output of the data association algorithm for solving the MTT problem is a set *ω*, which contains assigned tracks from the observations (*τ*_1_, *τ*_2_, …, *τ*_*K*_) and a false alarms set not assigned to any track (*τ*_0_). For simplification in notation, we call the set of estimated tracks {*τ*_1_, *τ*_2_, …, *τ*_*K*_} as *ω*.

**Figure 13:**
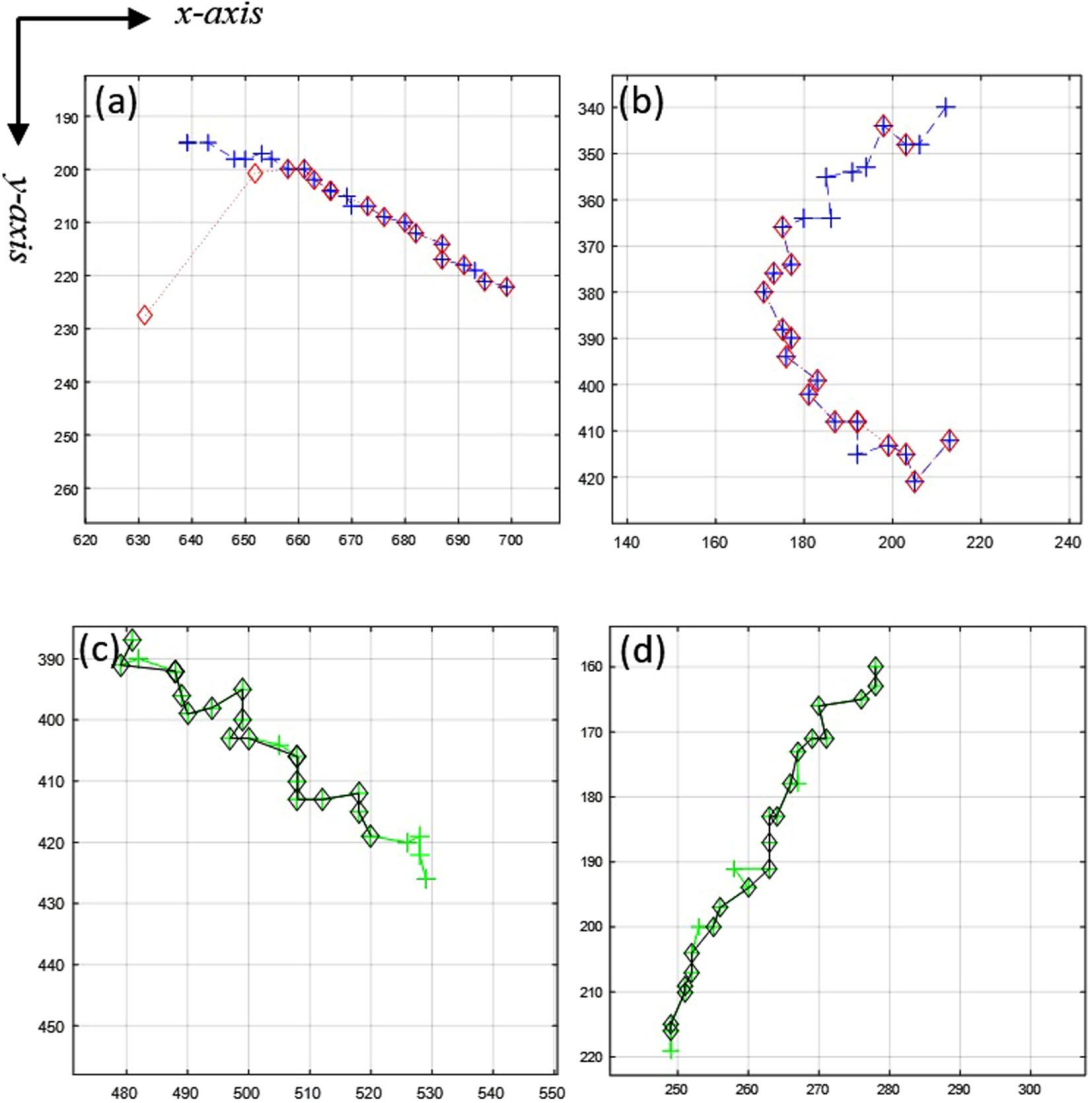
(**a**) Two tracks that did not match because of too much average distance between points. The blue cross markers show the ground-truth and the red diamonds show the estimated track. (**b**) Actually, there are three tracks: two tracks are separate estimated tracks and one is the ground truth. (**c**) Two matched tracks in which the green cross markers show the ground-truth and the black diamonds show the estimated track. There are certain missing observations in the track path. (**d**) Another matched track pair. It should be noted that the scales of the figures may vary and for precise investigation, the axes’ numbers must be taken into account.

As mentioned in many studies like^52^, one key problem for evaluating any MTT algorithm (independent of the algorithm and its properties) is how to optimally pair the set of estimated tracks *ω* and the set of ground-truth tracks ${\mathscr{G}}$. There are two problems: firstly, matching the tracks, and secondly, matching the points within the tracks. For solving the first problem, we should first solve the second problem. For the best matching between track pairs, we should calculate a distance between each track in *ω* and ${\mathscr{G}}$. Finding the distance between tracks which needs matching the points within the tracks (solving the second problem) is like Equation (11) and as described there, some dummy points were added to compensate for the missing points in the tracks in *ω*. After a distance calculation, a distance matrix is made. From the distance matrix and using Munkres algorithm^52,53^, we can optimally assign the tracks in *ω* to the tracks in ${\mathscr{G}}$. There might be some tracks in *ω* and ${\mathscr{G}}$ without any match, either because of being too far from any tracks in the other set or as the number of tracks in *ω* and ${\mathscr{G}}$ mismatch. If the first and last point of two tracks are farther than 25-pixels then their matching should be rejected. The maximum total number of missing observations is $\bar{d}$ which was set to 5, so in the worst case, having initially (or finally) 5 consecutive missing observations and assume average spermatozoa movement as 5 pixels (actually it is 4 pixels but we add an extra margin about 25%), then the distance between the first and last point should not exceed 25-pixels. Hence if the distance between the first and last points of the two tracks exceeds 25 pixels or average distance between points of two tracks exceeds 50 pixels, then they could not be labeled as matched. Matching tracks together with any “cost” (or distance) is not the goal of the MTT, because if there exist some spurious tracks due to false alarms (which is the case when the SNR is low and the false alarm rate is high), matching them to some ground truth tracks which did not really tracked, is not a correct approach. We define *n*_*C*_ as the number of correct associations (matched track between *ω* and ${\mathscr{G}}$) made by any algorithm. Figure 13 shows certain tracks that matched and certain tracks that did not match.

For representing the performance of the developed algorithm on the dataset, there must be some criteria to compare the results with other well-known algorithms in this context. There is a performance measure called *F*_1_, which has been used for the evaluation of methods like the data association in record matching^62^. The *F*_1_ measure is based on two other measures: precision and recall. The definition of these two measures is as follows:

- Precision: $$P=\frac{{n}_{C}}{\Vert \omega \Vert }$$
- Recall: $$R=\frac{{n}_{C}}{\Vert {\mathscr{G}}\Vert }$$

Now, based on these two measures, the *F*_1_ measure is defined as the harmonic mean between them

22 $${F}_{1}=\frac{2RP}{R+P}=\frac{2{n}_{C}}{\Vert \omega \Vert +\Vert {\mathscr{G}}\Vert }$$

*R* and *P* are related to effectiveness of the algorithm and so the higher the *F*_1_ measure, the more effective the algorithm^63^.

There is also another standard measure for precision in the track’s path: RMSE; this is a measure of precision in *correct associated tracks*. RMSE is calculated as follows:

23 $$RMSE({\tau }_{i},{\tau }_{j})=\sqrt{\frac{{\sum }_{t=1}^{T(i)}{({x}_{t}^{i}-{x}_{t}^{j})}^{2}+{({y}_{t}^{i}-{y}_{t}^{j})}^{2}}{T(i)}}$$

In Equation (23), *T*(*i*) is the length of the tracks. Finally, for a comparison between different algorithms, the mean of all the RMSEs in the whole dataset is calculated as an important measure of the algorithm performance.

Many algorithms have been introduced in “introduction” Section for solving MTT problem, and among all of them, two methods were selected for the implementation and comparison of the HDBN on the current dataset. First, MHT, as a MAP solution to the MTT problem, was chosen. The MATLAB implementation of MHT was used^64^. The maximum track tree depth was 5 in the *k*-best hypothesis; *k* was set to 6, and the maximum number of leaves after pruning was set to 5. Another method that was implemented in MATLAB was NNF as a standard method in MTT.

PDAF and JPDAF are also well-known algorithms for solving the MTT problem, but its inability to start and end a track automatically^29^ is a great disadvantage in high-density problems like spermatozoa tracking; for this shortcoming, these algorithms were not considered for implementation and comparison in the current study.

In “Design HDBN for data association” Subsection, the maximum number of consecutive missing observations called $\bar{d}$ was introduced and it was emphasized that if this threshold was passed, the track should be terminated. For determining this value, we must know the effects of this parameter mathematically and statistically. The probability of observing a specific target at least once in exactly $\bar{d}$ consecutive frames is a function of $\bar{d}$ as follows:

24 $${p}_{{\det }}(\bar{d})=1-{(1-{p}_{d})}^{\bar{d}}$$

If we want ${p}_{{\det }}(\bar{d})\ge \pi$, then from (24), we should have $\bar{d}\ge \,\mathrm{log}\,(1-\pi )/\,\mathrm{log}\,(1-{p}_{d})$. Now, if we set *π* = 0.99 and the average over *p*_*d*_, which yields 0.67, then we should have $\bar{d}\ge 5$. So, in all the implemented methods, $\bar{d}$ was set to 5.

**Figure 14:**
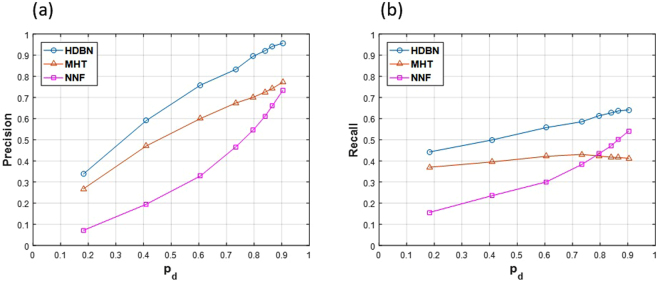
(**a**) Mean precision over all 36 image sequences and the (**b**) mean recall over all 36 image sequences plotted against *p*_*d*_ for the three methods.

**Figure 15:**
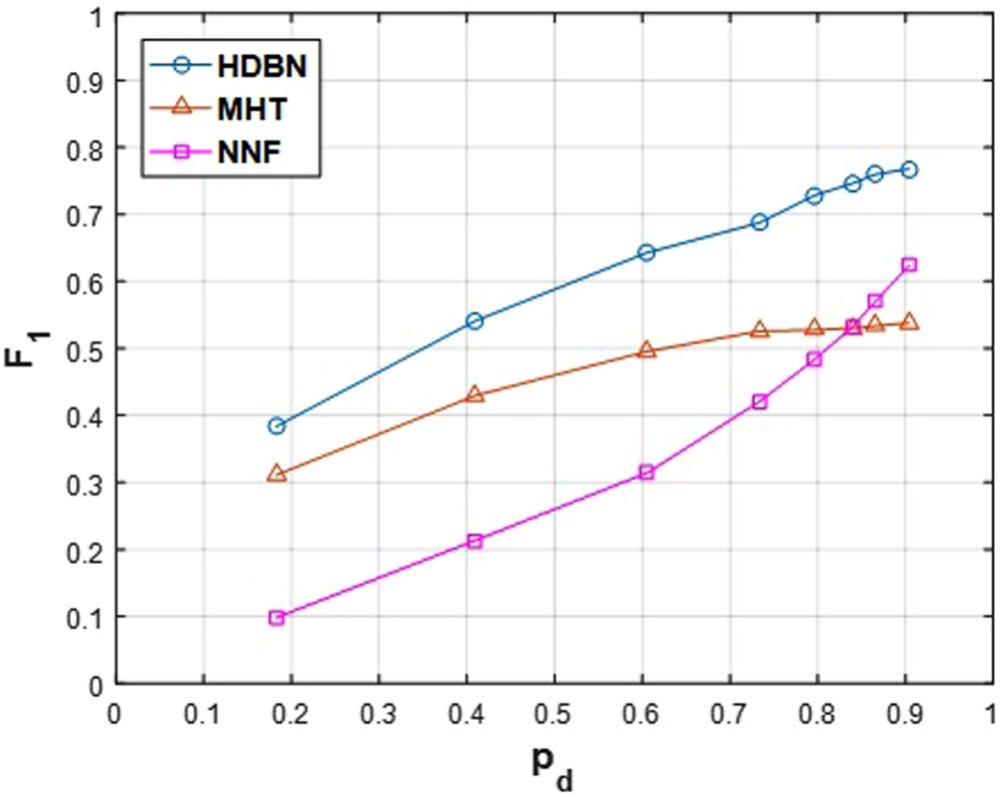
Mean *F*_1_ measure over all the 36 image sequences plotted against *p*_*d*_ for the three methods.

All the implemented algorithms were run on the dataset. HDBN was implemented with LOOCV. For a greater comparison between different conditions, the *β*_*max*_ parameter was swept; so, different variables for *p*_*d*_ and *FAR* were prepared according to curves in Fig. 6. Firstly, Precision and Recall were computed (Fig. 14), and then, the *F*_1_ measure was calculated based on these two measures (Fig. 15). The superiority of the proposed HDBN method could be observed from the plotted curves. The MHT algorithm precision shows a greater increase against detection probability than its recall. The NNF algorithm has the steepest growth against the increase of detection probability. Note that in these figures the sweeping *p*_*d*_ is alongside *FAR*, because these two parameters are connected together and acquired as a result of the segmentation algorithm.

**Figure 16:**
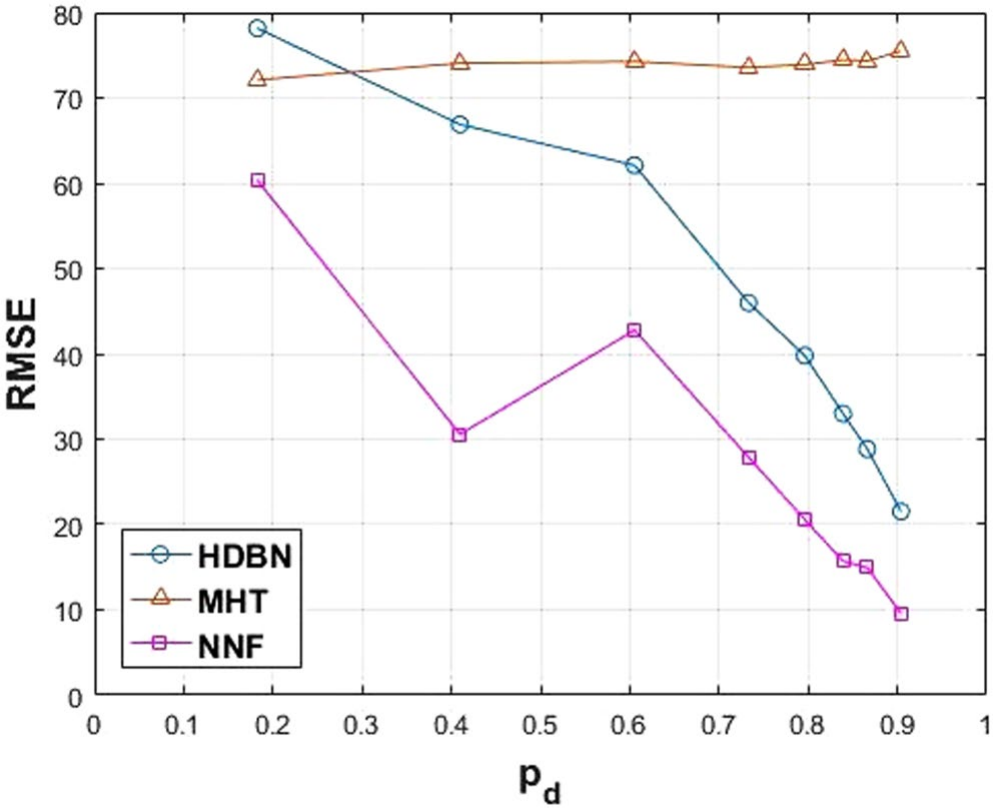
Mean *NRMSE* measure over all the 36 image sequences plotted against *p*_*d*_ for the three methods.

Another measure worth mentioning here is RMSE, which is a measure of how close the trajectories of the tracks have been to the ground-truth. The RMSE curve is plotted in Fig. 16 for each method. Note that RMSE of NNF is lower than the proposed method for high values of *p*_*d*_, which may be because of the nature of NNF method that selects the nearest observation to the last point of the track. This approach may fail when there is too much noise or clutter. All the results are also summarized in Table 3 and the superiority of HDBN can be confirmed in a majority of the cases.

**Table 3:** Summary of all the results with their standard deviation (the best results are in bold font).

*p*_*d*_	Measures	Method		
		NNF	MHT	HDBN
0.1825	Precision	0.0714 ± 0.0565	0.2680 ± 0.2032	**0.3382** ± **0.2004**
	Recall	0.1563 ± 0.1028	0.3700 ± 0.2293	**0.4422** ± **0.1788**
	*F*_1_ measure	0.0980 ± 0.0646	0.3108 ± 0.1555	**0.3833** ± **0.1772**
	RMSE	**60.348** ± **59.105**	72.099 ± 17.396	78.154 ± 23.973
0.4097	Precision	0.1950 ± 0.1400	0.4706 ± 0.2787	**0.5908** ± **0.2281**
	Recall	0.2358 ± 0.1507	0.3951 ± 0.2007	**0.4993** ± **0.1687**
	*F*_1_ measure	0.2135 ± 0.1143	0.4296 ± 0.1814	**0.5412** ± **0.1879**
	RMSE	**30.570** ± **29.524**	74.076 ± 15.081	66.885 ± 18.418
0.6053	Precision	0.3285 ± 0.1892	0.6000 ± 0.3066	**0.7583** ± **0.2059**
	Recall	0.3008 ± 0.1605	0.4217 ± 0.1694	**0.5577** ± **0.1657**
	*F*_1_ measure	0.3140 ± 0.1575	0.4953 ± 0.1868	**0.6427** ± **0.1796**
	RMSE	**42.775** ± **68.327**	74.264 ± 14.597	62.102 ± 20.563
0.7331	Precision	0.4658 ± 0.2454	0.6737 ± 0.2738	**0.8327** ± **0.1746**
	Recall	0.3827 ± 0.1993	0.4307 ± 0.1522	**0.5851** ± **0.1713**
	*F*_1_ measure	0.4202 ± 0.2097	0.5254 ± 0.1657	**0.6873** ± **0.1744**
	RMSE	**27.869** ± **37.791**	73.564 ± 11.679	45.971 ± 25.523
0.7961	Precision	0.5453 ± 0.2267	0.7006 ± 0.2672	**0.8946** ± **0.0879**
	Recall	0.4342 ± 0.1824	0.4230 ± 0.1573	**0.6139** ± **0.1516**
	*F*_1_ measure	0.4835 ± 0.1995	0.5275 ± 0.1676	**0.7281** ± **0.1383**
	RMSE	**20.576** ± **25.020**	73.901 ± 11.691	39.802 ± 25.157
0.8390	Precision	0.6102 ± 0.1829	0.7254 ± 0.2457	**0.9209** ± **0.0676**
	Recall	0.4704 ± 0.1582	0.4175 ± 0.1541	**0.6268 ± 0.1504**
	*F*_1_ measure	0.5312 ± 0.1678	0.5300 ± 0.1587	**0.7459** ± **0.1325**
	RMSE	**15.698** ± **14.479**	74.446 ± 8.253	32.994 ± 20.522
0.8662	Precision	0.6617 ± 0.1489	0.7426 ± 0.2360	**0.9423** ± **0.0522**
	Recall	0.5020 ± 0.1438	0.4164 ± 0.1563	**0.6367** ± **0.1430**
	*F*_1_ measure	0.5709 ± 0.1462	0.5336 ± 0.1573	**0.7599** ± **0.1217**
	RMSE	**14.953** ± **12.816**	74.335 ± 8.552	28.846 ± 16.237
0.9041	Precision	0.7342 ± 0.1251	0.7733 ± 0.1919	**0.9566 ± 0.0482**
	Recall	0.5420 ± 0.1515	0.4123 ± 0.1517	**0.6414** ± **0.1547**
	*F*_1_ measure	0.6236 ± 0.1445	0.5378 ± 0.1698	**0.7679** ± **0.1327**
	RMSE	**9.568** ± **7.322**	75.481 ± 5.991	21.514 ± 9.869

Superiority of the HDBN in accuracy arose from predicting the probability for each observation in a correct structure as well as by the use of a prior knowledge of spermatozoa movement patterns. The calculation for the next point of a track and selection among many observations is the fundamental key toward achieving a good result in data association. Based on the reality that there is randomness in spermatozoa movements, there might be some patterns; the HDBN tried to discover the most likely patterns related to the current track being tracked for current time step. The most likely patterns will suggest some points from the observations set and rank each with a probability. Finally, selecting the most probable point among all the points advances the track to next time step.

**Figure 17:**
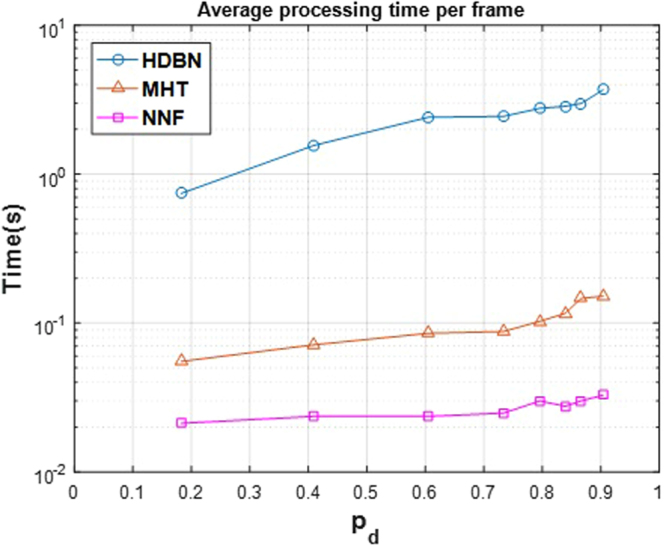
Average time complexity for processing a single frame (averaged overall in 36 image sequences) plotted against *p*_*d*_ for three methods. It should be noted that for a better representation, the Y-axis is in the logarithmic scale.

Time complexity of an algorithm is also an important measure. In Fig. 17, the average time needed for processing a single frame is plotted for each method. It is obvious that the time complexity grows with *p*_*d*_ because as the detection probability increases, more targets, and as a result, more tracks are detected and are going to be completed. The algorithms were run on a Windows ® based Laptop with Intel ® Core™ i7-3630QM CPU with 16GB of memory installed on it. All algorithm was implemented in MATLAB ® R2016a. Time complexity of the proposed method is high in comparison to the other methods, but it is still acceptable (about a few seconds per frame). One reason for this high time complexity is calculation of $p({\gamma }_{t+1}^{i}|{{\boldsymbol{Z}}}_{t})$, which directly depends on the size of ***Z***_*t*_. In the current study, the average size of ***Z***_*t*_ was about 1,600 samples. Reducing the samples will reduce the time complexity, but may alter (and usually, degrade) the performance. The number of samples that can be removed from the dataset so as to get approximately the same result (discussed in Conclusion section) can be investigated in the future.

## Conclusion

In this paper, a method based on HDBN has been presented. A new HDBN model was designed based on Softmax CPD for inference and solving the MTT problem. In the presented model, exploiting the manually extracted dataset as a source of information for track guidance has been introduced. For the best compatibility between tracks, track normalization has been introduced in polar representation as a practical tool for the better usage of the manually extracted dataset.

For the evaluation of the developed algorithm, a currently up-and-running CASA research system has been used for recording many samples, and then, the tracks from the recorded samples have been extracted manually for building a ground-truth and a dataset. The dataset of the current study is quite large (there were more than 1,650 spermatozoa tracks in the dataset); so, the final results were more reliable.

The segmentation was performed with a control parameter (maximum blob area) and by sweeping that, the various detection probability and false alarm rates were yielded. Different detection probabilities (as well sd false alarm rates) were used each time to run different data association algorithms; so, we can finally compare the performance of each algorithm based on a common observation step. Thus, we have compared the data association qualities in each tracking algorithm.

Finally, the developed method was implemented and tested on the dataset and compared to other well-known algorithms, such as MHT and NNF, for solving MTT. The results showed the superiority of the developed algorithm in many measures, including precision, recall, *F*_1_ measure, and *RMSE*. The superiority of the HDBN in accuracy came from predicting the probability for each observation in a correct structure and also by use of a prior knowledge of spermatozoa movement patterns. The calculation of the next point of a track and selection among many observations is the fundamental key for achieving a good result in data association. Based on the fact that there is also randomness in spermatozoa movements, there might be some patterns; the HDBN tried to discover the most likely patterns related to the current track being tracked for the current time step. The most likely patterns will suggest certain points from the observations set and rank each with a probability. Finally, selecting the most probable point among all the points in related gating selects the points for the track and the algorithm proceeds in the next time step. Gating was used for reducing the process time and avoiding the calculation of probability for unlikely points which were too far to be considered as the following points of the current track.

The only issue with the developed algorithm is that the process of calculating the probability distribution over all the samples of the dataset is time-consuming. However, because the MTT problem in spermatozoa tracking need not to be in real-time in most cases, this issue is not a bottleneck. Processing each frame in a few seconds is an acceptable speed for many applications, including fertility research. In other real-time applications, there must be some modification to the algorithm, e.g., reducing the size of dataset for calculating the probability or selecting some more relevant tracks so that the computation time is reduced.

The current study can be extended in several ways in future work:

- The observation studied in this paper was limited (although enough for the investigation of the developed algorithm); this step and its effects on the consequent steps can be broadly studied separately, both as some new segmentation methods in this field or by the means of simulations and by artificially manipulating *p*_*d*_, *p*_*m*_, and *FAR* on the manually extracted dataset.
- Certain heuristic methods have been focused on recently and studied for solving the MTT problem; these include Markov Chain Monte Carlo (MCMC) and sampling methods like the Metropolis–Hastings (MH) sampling algorithm. Testing these algorithms on the dataset could lead to valuable information and a comparison to the other methods in different scenarios.
- The time complexity of the algorithm is high and it could be reduced by optimizing the set of dataset used for the next observation selection. The current dataset is large and results in a huge amount of computation tasks to sweep all the samples. Retaining the same performance, there could be other methods for using the dataset in a different order and by reducing its size, we can achieve better performance in time complexity. This may be done by categorizing the movements and using the most informative samples only, and discarding repeated patterns that are similar to each other and do not add much more information to the system.
- Testing the developed algorithm on the other recorded datasets as well as on some synthetically generated data like^65,66^ is another benchmark for further testing and confirming the achieved results.
